# *Asterias forbesi*-Inspired SERS Substrates for Wide-Range Detection of Uric Acid

**DOI:** 10.3390/bios14010008

**Published:** 2023-12-22

**Authors:** Hyunjun Park, Kyunghwan Chai, Woochang Kim, Joohyung Park, Wonseok Lee, Jinsung Park

**Affiliations:** 1 Department of Biomechatronic Engineering, College of Biotechnology and Bioengineering, Sungkyunkwan University, Suwon 16419, Republic of Korea; guswns1105@g.skku.edu (H.P.); kyungh2012@g.skku.edu (K.C.); wkim91@skku.edu (W.K.); parkjoodori@skku.edu (J.P.); 2 Department of Electrical Engineering, Korea National University of Transportation, Chungju 27469, Republic of Korea

**Keywords:** uric acid, wide-range detection, bioinspired, gold nanoparticles, surface-enhanced Raman scattering

## Abstract

Uric acid (UA), the final metabolite of purine, is primarily excreted through urine to maintain an appropriate concentration in the bloodstream. However, any malfunction in this process can lead to complications due to either deficiency or excess amount of UA. Hence, the development of a sensor platform with a wide-range detection is crucial. To realize this, we fabricated a surface-enhanced Raman spectroscopy (SERS) substrate inspired by a type of starfish with numerous protrusions, *Asterias forbesi*. The *Asterias forbesi*-inspired SERS (AF-SERS) substrate utilized an Au@Ag nanostructure and gold nanoparticles to mimic the leg and protrusion morphology of the starfish. This substrate exhibited excellent Raman performance due to numerous hotspots, demonstrating outstanding stability, reproducibility, and repeatability. In laboratory settings, we successfully detected UA down to a concentration of 1.16 nM (limit of detection) and demonstrated selectivity against various metabolites. In the experiments designed for real-world application, the AF-SERS substrate detected a broad range of UA concentrations, covering deficiencies and excesses, in both serum and urine samples. These results underscore the potential of the developed AF-SERS substrate as a practical detection platform for UA in real-world applications.

## 1. Introduction

Uric acid (UA), primarily synthesized in the liver, is an organic compound produced endogenously from purine metabolism in the human body. It circulates through the bloodstream and is excreted in urine via kidneys [1]. UA functions as a biomarker for oxidative stress, and the changes in its concentration are associated with various physiological and pathological conditions [2]. Generally, the normal concentration range of UA in the blood is approximately 200 to 500 μM [3,4], and deviations from this range can lead to complications. If UA levels in the blood drop below the normal range (a condition known as hypouricemia), then the risk of acute kidney injury can significantly increase after intense physical activity. Additionally, low UA levels in the blood are associated with renal hypouricemia, which is linked to kidney stones due to an increased level of urinary UA, correlating with the impaired tubular reabsorption of UA [5]. Conversely, heightened UA levels, referred to as hyperuricemia, can result in the formation of urate crystals, which accumulate in joints, cartilage, and adjacent tissues, giving rise to conditions such as gout [6,7]. Additionally, hyperuricemia is associated with inflammatory diseases, kidney issues, and hypertension [8,9,10,11]. Therefore, detecting UA concentrations outside the normal range in both blood and urine is crucial for assessing health status and diagnosing diseases.

Due to the critical importance of detecting UA levels, tests for UA in blood and urine are performed via standard procedures in hospitals. However, these tests have limitations, including the need for periodic sample collection and intricate preparation procedures, and these constraints have driven the development of various UA detection methods over the past decades [12,13,14]. Techniques involving electrochemistry [15,16], fluorescence [17], and spectroscopy [18,19] have been widely employed in UA detection. In particular, electrochemical detection methods are prevalent because of their effectiveness in UA detection. However, UA produces a redox signal similar to dopamine and ascorbic acid, which may cause selectivity problems [20,21,22]. Recently, spectroscopy based on surface-enhanced Raman scattering (SERS) has garnered significant attention in the biosensing field due to its remarkable sensitivity and exceptional selectivity [23,24]. However, the current SERS-based platforms still face limitations in detecting UA in both deficiency and excess ranges [25]. To overcome this challenge, it is crucial to design nanostructures with high sensitivity and extensive surface areas. Fabricating nanostructures with numerous nanogaps and forming SERS substrates with broad surface areas capable of accommodating high concentrations of analytes are essential for addressing this limitation.

In this study, we drew inspiration from the morphology of *Asterias forbesi*, commonly known as the Forbes Sea Star, to enhance the performance of the SERS substrate. *Asterias forbesi* exhibits numerous protrusions on its torso and legs, resembling structures in which the SERS effect can be maximized by creating nanoscale hotspots. To replicate this structure, we strengthened the SERS effect by fabricating small-scale gold nanoparticles (GNPs) in a gold–silver bimetallic structure, imitating starfish legs and the protuberances of *Asterias forbesi*. This design maximized the SERS effect by increasing the adhesion area with UA and promoting hotspot creation. The *Asterias forbesi*-inspired SERS (AF-SERS) substrate demonstrated an outstanding SERS effect; this result was further confirmed through electromagnetic field simulations. Using the AF-SERS substrate, UA was detected across a wide range of samples, including serum and human urine. Moreover, the substrate enabled the specific identification and detection of UA among various endogenous metabolites. These findings showcase the promising potential of the developed AF-SERS substrate as a sensor platform not only for UA but also for various targets in real-world field settings.

## 2. Experimental Section

### 2.1. Chemical Agents

The reagent for SERS substrate fabrication and SERS performance analysis (sodium citrate, chloroauric acid (HAuCl_4_), potassium dicyanoargentate (KAg(CN)_2_), Rhodamine 6G(R6G)), UA, selective experimental group (L-ascorbic acid, hydrochloride dopamine, L-cysteine, D-(+)-glucose, creatine), and human serum and sigmatrix urine diluent (artificial urine) were purchased from Sigma Aldrich (St. Louis, MO, USA). A glass slide was purchased from Paul Marienfeld GmbH & Co. KG (Lauda-Königshofen, Germany). UA solution was generated by dissolving it in deionized (DI) water, and the selectivity analysis group was prepared in the same manner. All glassware was washed with piranha solution in concentrated sulfuric acid (98% *w*/*v*) and hydrogen peroxide (30% *w*/*v*) prior to use. All solutions were prepared using Millipore DI water with a resistivity of at least 18.2 MΩ cm at 25 °C. Additionally, all target samples for final substrate production were reacted with GNP at a 1:1 ratio based on volume.

### 2.2. Uric Acid Detection Strategy Using AF-SERS Substrate

**Scheme 1:**
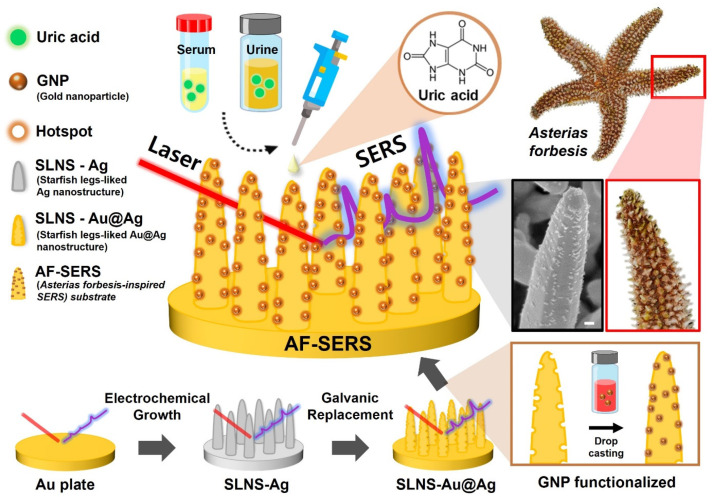
Schematic of Raman spectroscopic detection of UA derived from serum and urine using an *Asterias forbesi*-inspired SERS substrate.

To develop a sensor capable of simultaneously detecting UA deficiencies and excesses outside the normal range in both blood and urine, a SERS structure with a broad surface area is crucial. This type of structure ensures an effective response even at high UA concentrations [26]. Therefore, we developed a detection strategy based on a SERS substrate inspired by *Asterias forbesi*, commonly known as Forbes Sea Star (Scheme 1). Initially, an electrochemical method was employed to produce a silver nanostructure resembling the legs of a starfish. This was achieved by reducing silver using the KAg(CN)_2_ solution on the surface of the gold plate through overpotential [27]. However, various substances present in serum and urine can promote silver oxidation, leading to a decline in the intensity of the SERS signal [28]. To address this, we employed galvanic substitution for reducing gold ions using an HAuCl_4_ solution, thereby coating the surface with gold while simultaneously inducing surface roughening. Subsequently, through the integration of gold-coated silver nanostructures with GNPs, a nanostructure resembling the arms of *Asterias forbesi* was successfully fabricated. This was confirmed through SEM images, demonstrating that the developed structure was similar to the spikes in *Asterias forbesi*. These nanostructures exhibited numerous hotspots, a wide surface area facilitated by high Z-axis and particle characteristics, and a bimetallic enhancement effect, which combined the effective plasmonic properties of silver with the chemical stability of gold, ensuring a strong SERS effect [29]. Through these enhancements, the SERS substrate effectively detected UA in serum and urine samples.

### 2.3. Synthesis and Optimization of AF-SERS Substrate

Starfish-leg-like Ag nanostructure (SLNS-Ag) and starfish-leg-like Au@Ag nanostructure (SLNS-Au@Ag) were fabricated using the methods described in previous studies [30,31]. Briefly, bare Au plate was fabricated using an E-beam evaporator, and SLNS-Ag was produced through the reduction in KAg(CN)_2_ by employing chronoamperometry. Subsequently, 50 μL of 0.2 mM HAuCl_4_ was placed in a polydimethylsiloxane (PDMS) mold for 30 min to induce galvanic replacement, resulting in the fabrication of SLNS-Au@Ag.

GNPs were synthesized using the Turkevich method [32]. For the synthesis of GNP, 1 mL of 1 wt% HAuCl_4_ solution and 5 mL of 1 wt% sodium citrate solution were added to 100 mL of DI water and heated at 100 °C for 1 h with vigorous stirring. Afterward, the target substance to be detected was mixed with the synthesized GNP solution in a 1:1 ratio and dropped onto the SLNS-Au@Ag substrate to complete the production of the AF-SERS substrate. For sensitive and precise measurements, each AF-SERS substrate was exclusively used for a single sample, ensuring that none was reused to maintain the highest standards of accuracy.

### 2.4. SEM- and FEM-Based Simulation Data

The morphologies of SERS substrates were observed via field-emission scanning electron microscopy (FE-SEM; JSM-IT800, JEOL, Tokyo, Japan). SEM images were obtained at 15 kV HV at different magnifications.

Finite elements method (FEM)-based plasmonic simulations of the optimal SERS substrate analysis were performed using the electromagnetic wave module of COMSOL Multiphysics 6.0. The structures of the nanopillar SERS substrate (Au plate, SLNS-Ag, SLNS-Au@Ag) and nanopillar-combined GNP were based on the SEM images. The time harmonic maxwell equation with boundary conditions was calculated for plasmonic SERS substrates. The simulation was vertically projected onto the surface of the SERS substrate with an incident light of 785 nm. The refractive index and simulation function were referenced from a previous study [26], and RGB values were used to analyze the FEM simulation results using Image J software(Image J v.1.53).

### 2.5. SERS Analysis

SERS spectra were analyzed using Raman spectroscopy (In Via Reflex, Renishaw, Wotton-under-Edge, UK). The laser was focused using a 100× objective lens (Leica DM2700 M, DEU, and Renishaw Centrus Detector, GBR), and the diameter of the spot of the laser beam was approximately 0.4 µm. All SERS spectra were measured using a 785 nm laser with a power of 5.05 mW after 1 s exposure and 10 accumulations.

The Raman spectra of R6G were measured in the range of 567–1675 cm^−1^, whereas those of UA and the selective experimental group were measured in the range of 497–1622 cm^−1^.

### 2.6. Optimization of SERS Substrate

To analyze the optimal SERS substrate, 5 μL droplets of 100 μM R6G were reacted on the following prepared substrates: Au plates, SLNS-Ag, and SLNS-Au@Ag. Additionally, to evaluate the SERS performance of GNP-functionalized substrates, 5 μL droplets of R6G and GNP mixed in a 1:1 volume ratio were applied to each substrate (Au plate, SLNS-Ag, and SLNS-Au@Ag).

To optimize the GNP concentration, the synthesized GNPs were precipitated via centrifugation (Labogene 1524, LABOGENE, Lillerød, Denmark) at 6500× *g* rpm (2669 g) for 30 min, and the supernatant was removed or more DI water was added to prepare GNPs at various concentrations (×0.2–×5). To optimize the quantity of GNPs, various concentrations of GNP solutions were prepared using the synthesized GNP stock solution (×1). Diluted GNP solutions were prepared by diluting the original solution fivefold (×0.2) and twofold (×0.5) using DI water. Concentrated solutions, obtained through centrifugation with concentrations twofold (×2) and fivefold (×5), were prepared for use. The resulting different concentrations of GNPs were mixed with R6G at a 1:1 ratio, and 5 μL droplets were reacted with SLNS-Au@Ag for subsequent SERS measurements.

Initially, a series of SLNS-Ag and SLNS-Au@Ag were prepared and exposed to a challenging 10× PBS (phosphate-buffered saline, 100 mM) environment. Subsequently, these particles were individually retrieved on specified days and thoroughly washed with ethanol (EtOH) and DI water. Afterward, 5 μL of GNP solution combined with 100 μM R6G in a 1:1 ratio were deposited onto the washed substrates containing SLNS-Ag and SLNS-Au@Ag. Subsequently, the variations in the Raman spectra of R6G on each substrate were measured daily.

### 2.7. Uric Acid Detection with AF-SERS SERS Sensors

All sample preparations involved mixing the sample with 1X GNP at a 1:1 volume ratio and subsequently applying a 5 μL droplet onto the GNP substrate, and samples were allowed to dry prior to SERS measurements.

The reproducibility and uniformity analyses of the fabricated AF-SERS substrate were conducted using a 100 μM UA solution. Reproducibility analysis entailed reacting the UA of the same concentration with six independently prepared substrates to measure the Raman intensity. Uniformity analysis involved measuring the Raman intensity of UA at 50 randomly selected spots on the prepared substrates.

In laboratory conditions, UA detection was performed in the concentration range from 1 nM to 1 mM. The selectivity of the UA sensor was evaluated using various biological fluid materials, including dopamine, L-ascorbic acid, D-(+)-glucose, L-cysteine, and creatine, each at a concentration of 1 mM.

The actual application of the UA sensor involved the use of 10% diluted human serum and artificial urine samples. Various concentrations of UA ranging from 1 μM to 1 mM were prepared using 10% diluted human serum. Additionally, using artificial urine samples, we prepared samples with UA concentrations within the normal range, representing 300 μM, as well as samples indicating UA deficiency with a concentration of 30 μM and excess conditions with a concentration of 3 mM. The Raman measurement conditions were maintained consistent with those used for Raman analysis.

## 3. Results and Discussion

### 3.1. Characterization of AF-SERS Substrate

**Figure 1:**
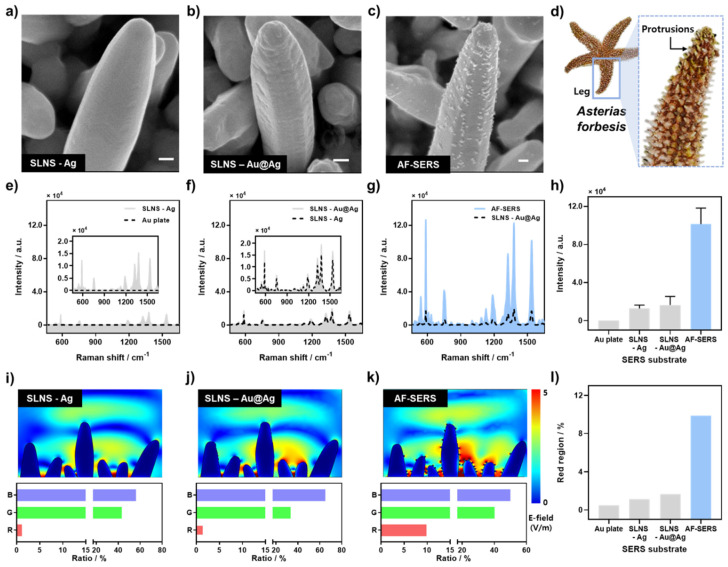
SEM images of (**a**) SLNS-Ag, (**b**) SLNS-Au@Ag, and (**c**) AF-SERS substrate (scale bar: 100 nm). (**d**) Image of the legs and protrusion structures of the *Asterias forbesi*. Raman spectrum comparison data for 100 μM R6G, a Raman emitter: (**e**) Bare Au plate and SLNS-Ag substrate (Gray scale: SLNS-Ag, Black dotted line: Au plate), (**f**) SLNS-Ag and SLNS-Au@Ag substrate (Gray scale: SLNS-Au@Ag, Black dotted line: SLNS-Ag), and (**g**) SLNS-Au@Ag and AF-SERS substrate (Blue scale: AF-SERS, Black dotted line: SLNS-Au@Ag), respectively. (**h**) Raman intensities at 1508 cm^−1^, a specific peak of R6G on various SERS substrates (Au plate, SLNS-Ag, SLNS-Au@Ag, and AF-SERS, respectively). FEM-based electromagnetic simulation results and RGB value graph for each area of (**i**) SLNS-Ag, (**j**) SLNS-Au@Ag, and (**k**) AF-SERS substrate. (**l**) Ratio of the red region (in (**i**–**k**)) for each substrate.

To confirm the characteristics of the AF-SERS substrate, various analyses, including morphology, Raman intensity, and electromagnetic field simulations, were conducted (Figure 1). Initially, SEM was employed for morphological analysis. Figure 1a confirms the successful fabrication of starfish-leg-shaped silver nanostructures (SLNS-Ag) using electrochemical methods. Subsequently, after galvanic replacement, the surface of SLNS-Ag was replaced with gold, resulting in SLNS-Au@Ag. This replacement led to a darker appearance of the substrate surface (Figure S1), and SEM images revealed a slightly roughened surface compared with the smooth SLNS-Ag surface (Figure 1b). This roughening was attributed to the deposition of gold in the areas where silver was displaced. The displacement reaction is described by the following equation [31]:

Anode Reaction: 3Ag → 3Ag^+^ + 3e^−^

Cathode Reaction: AuCl4^−^ + 3e^−^ → Au + 4Cl^−^

Finally, the SLNS-Au@Ag substrate functionalized with GNP assumed a structure similar to *Asterias forbesi* (Figure 1c,d). Figure S2 further validates the successful attachment of galvanic and gold nanoparticles through EDS analysis, which identifies the components of each Au and Ag. Additionally, the low-magnification SEM images for each condition are shown in Figure S3. Following that, we conducted Raman intensity analysis using R6G, a representative Raman indicator, based on the morphology observed at each step. R6G is a Raman indicator with fluorescent characteristics, and in order to minimize the impact of fluorescence in Raman analysis, we selected a laser with a wavelength of 785 nm [33]. When using the SLNS-Ag substrate as opposed to a gold plate, enhanced Raman signals at the characteristic peak of R6G (1508 cm^−1^) were observed [34], attributed to the hotspots created by the nanostructures (Figure 1e). Typically, in a Raman substrate utilizing silver, the intrinsic Raman peak of silver appears around 1000 cm^−1^. However, in Figure 1e, the Raman intensity attributed to the SERS effect of the Raman indicator, R6G, is relatively high, making it challenging to distinguish the Raman peak of silver, which appears at a much lower value. As seen in Figure S4, when the Raman intensity scale is reduced for closer examination, it is confirmed that, unlike the case with the Au plate, a Raman signal around 1000 cm^−1^ is observed in the SLNS-Ag substrate composed of silver [35]. SLNS-Au@Ag exhibited a slight enhancement in Raman intensity compared with SLNS-Ag, attributed to the gold–silver bimetallic effect resulting from the coexistence of gold and silver (Figure 1f) [36]. When evaluating the SERS intensity using the AF-SERS substrate, a significant amplification in the R6G SERS signal was observed (Figure 1g). This outcome might be anticipated solely due to GNP. However, when only GNPs were employed, the performance was inferior compared to when the AF-SERS was employed (Figure S5). These results suggest a synergistic effect between the structures with excellent Z-axis characteristics and nanoparticles. For the analysis of the Raman intensity of each substrate, emphasis was placed on 1508 cm^−1^, representing the peak value, which manifests the most conspicuous concentration change among the Raman peaks of R6G. Figure 1h presents a bar graph illustrating the average and variance in the Raman intensity at 1508 cm^−1^, facilitating a quantitative assessment of the Raman intensity for each substrate. The Raman intensity of the AF-SERS substrate surpassed that of the SLNS-Au@Ag substrate by more than 6.2 times, underscoring its outstanding SERS performance. These findings emphasize the highly effective characteristics of *Asterias forbesis*-inspired nanostructures as superior SERS substrates. Notably, the presence of smaller scale nanoparticles, in contrast to a singular metal nanostructure, leads to an increased reaction surface area with the target and the generation of additional hotspots [37,38].

To validate these results, we conducted electromagnetic field simulations, designing the morphologies to resemble the SEM images of each state (Figure 1i–k). The electron density generated through the simulations is depicted using colors, with red indicating high intensity, green representing intermediate intensity, and blue indicating low intensity. Initially, on the Au plate, hotspot formation was minimal, and the red area was scarce (Figure S6). However, as the nanostructures formed at each stage, the electromagnetic field signal was enhanced, leading to an increased presence of the red region (Figure 1i–k). Although SLNS-Ag and SLNS-Au@Ag appeared similar, slightly higher electron density was observed in SLNS-Au@Ag. Remarkably, the AF-SERS structure exhibited a broader red region, particularly on the GNP surface, indicating enhanced hotspots due to the numerous nanogaps formed by particles. This contributed to the reinforcement of SERS intensity. Quantitative analysis revealed that the red region for AF-SERS was 497.32% larger than that for the SLNS-Au@Ag substrates (Figure 1l). Therefore, it can be inferred that AF-SERS demonstrated increased SERS intensity due to the abundant formation of hotspots in both the nanostructures and starfish-leg-shaped structures. Ultimately, through this validation, we confirmed consistency between experimental results and simulation outcomes, providing robust support for our claims.

### 3.2. Optimization and Sensor Performance of AF-SERS Substrate

**Figure 2:**
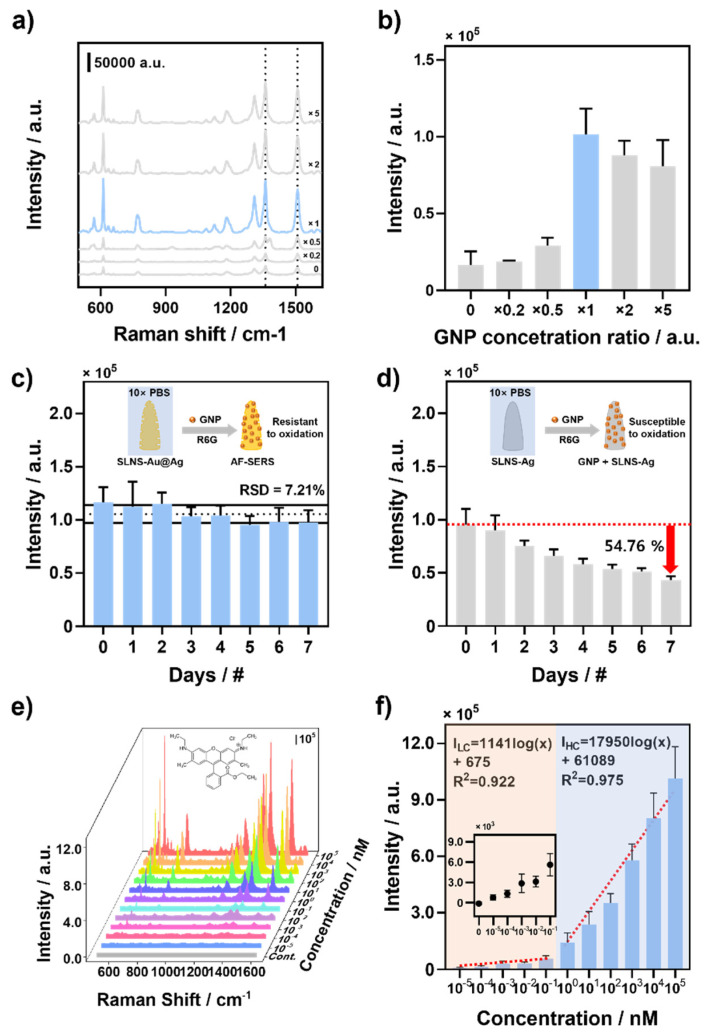
Optimization and SERS performance evaluation of AF-SERS with R6G. (**a**) SERS spectra of R6G depending on GNP concentration. (**b**) Measured values of the SERS peak intensities at 1508 cm^−1^ depending on the GNP concentration. Intensity at specific Raman peaks of R6G in (**c**) AF-SERS and (**d**) GNP + SLNS-Ag substrate measured under extreme conditions (10× PBS solution) for 7 days. (**e**) SERS sensitivity of R6G detection at various concentrations (10^–14^–10^–4^ M). (**f**) Measured values of SERS peak intensities at 1508 cm^−1^ depending on the R6G concentrations. Inset depicts the magnified SERS peak intensities of R6G at a low concentration (0–10^–10^ M).

The analysis of Raman intensity and simulations underscored the significant impact of the presence or absence of GNPs on the starfish-like nanostructure in determining SERS intensity. Consequently, optimizing the quantity of GNPs corresponding to the protuberances of *Asterias forbesi* was crucial for achieving optimal sensor performance. To explore this, we conducted an analysis of Raman intensity for R6G by varying the amount of GNP to identify the point of optimal effect. The quantity of GNP was adjusted by considering the amount of GNPs initially included in the synthesized GNP solution as 1, allowing for subsequent dilution or concentration to modify the relative GNP quantity. Detailed methods for this adjustment are described in the Experimental Section. Figure 2a presents the Raman spectrum data corresponding to the amount of GNP. As the amount of GNP increases from SLNS-Au@Ag without GNP, the Raman intensity values show an upward trend. To provide a detailed analysis of the Raman intensity of R6G under each condition, a bar graph was generated to represent the Raman intensity at 1508 cm^−1^ (Figure 2b). However, an excessive amount of GNPs can lead to aggregation and overlap, reducing hotspots and consequently diminishing the SERS effect [39,40]. Therefore, the highest Raman intensity was observed at the x1 concentration, confirming the importance of optimizing the amount of GNP for an effective response. Therefore, for subsequent experiments, the AF-SERS substrate fabricated under x1 conditions was used.

Hence, replacing silver with gold through galvanic substitution is a clever strategy to address the oxidation issue, as gold is more resistant to oxidation. This approach can significantly enhance the stability of the sensor, ensuring long-term consistent and reliable performance [41]. It provides a practical solution to overcome the limitations associated with silver structures, particularly when dealing with biomaterials. To assess the stability of the sensor, it was exposed to stringent environmental conditions (immersion in 10× PBS solution for 7 days), and Raman signals (R6G, 100 μM) were compared. The experiment involved using the AF-SERS substrate and GNP + SLNS-Ag substrate, where the galvanic replacement step was omitted. The results indicated that the AF-SERS substrate maintained a consistent Raman intensity over the entire 7-day period, with a relative standard deviation (RSD) value of 7.21% (Figure S7 and Figure 2c). The minimal changes observed over the 7-day period under rigorous environmental conditions imply that AF-SERS is also advantageous for long-term storage. In contrast, the control group, GNP + SLNS-Ag substrate, exhibited a noticeable reduction in Raman intensity at the same peak over time, resulting in a significant 54.76% decrease after 7 days (Figure S8 and Figure 2d). The AF-SERS substrate, coated with gold, demonstrated exceptional resistance to oxidation, effectively preventing a decrease in Raman intensity due to oxidation conditions.

To evaluate the sensitivity of the AF-SERS substrate, we utilized various concentrations of R6G, ranging from 100 μM to 10 fM. The specific Raman peak of R6G increased with higher concentrations (Figure 2e). For the quantitative analysis of Raman intensity, the Raman intensity at a specific R6G peak (1508 cm^−1^) was expressed using mean and variance (Figure 2f). Distinct linear behaviors were observed in both the low concentration (10^−5^–10^−1^ nM) and high concentration (10^2^–10^5^ nM). The linear equations for low and high concentration ranges are I_LC_ = 1141 × log(X) + 675 (R^2^ = 0.922) and I_HC_ = 17,950X + 61,089 (R^2^ = 0.975), respectively, where X represents the concentration of R6G. The limit of detection (LOD) was 1.395 fM, calculated using the formula LOD = 3.3 × standard deviation/slope. Based on these findings, we computed the analytical enhancement factor (AEF) for the AF-SERS substrate using the following formula [42]:

$$\mathrm{AEF}=\frac{I_{SERS}/C_{SERS}}{I_{OR}/C_{OR}}$$

Here, *I_SERS_* represents the SERS intensity; *I_OR_* denotes the SERS intensity observed on the bare substrate; *C_SERS_* stands for the concentration of the Raman indicator on the SERS substrate (i.e., LOD); and *C_OR_* is the concentration of the Raman indicator on the bare substrate. The computed analytical enhancement factor (AEF) for the AF-SERS was 3.658 × 10^11^, signifying enhancement in performance compared to other SERS substrates (Table S1). Consequently, the AF-SERS substrate demonstrated highly sensitive detection of R6G and successfully detected a wide concentration range. These findings suggest a promising potential of the developed substrate for the detection of UA and other analytes in future applications.

### 3.3. Performance Evaluation of AF-SERS Substrate with UA

**Figure 3:**
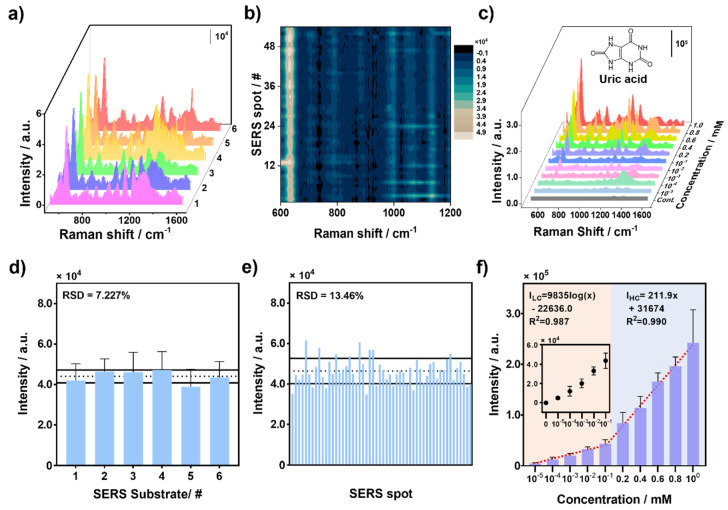
Efficiency and sensitivity analysis of the AF-SERS-based UA sensor. (**a**) R6G Raman spectra of six different AF-SERS substrates and (**b**) Heat map image of R6G Raman spectra at 50 random spots. (**c**) Raman spectra for UA detection at various concentrations on AF-SERS SERS substrate. Inset image is the molecular structure of UA. (**d**) Raman intensity at 1508 cm^−1^, which is the specific peak of R6G for each substrate. (**e**) Raman intensity measurements at 1508 cm^−1^ for 50 random data points extracted from heat map data. (**f**) Measured value of the SERS peak intensities at 640 cm^−1^ depending on the UA concentration. Inset image shows the results in the low concentration range, presented at an optimal scale.

Subsequently, the suitability of the AF-SERS substrate for UA detection was examined, considering the reproducibility between substrates, random spot reproducibility, and UA detection performance (Figure 3). The normal Raman spectrum of uric acid is depicted in the following figure, with band assignment displayed in Table S2. Initially, six different AF-SERS substrates were fabricated, and each substrate was reacted with a consistent 5 μL of 100 μM UA (Figure 3a). The specific Raman peak of UA occurred at 640 cm^−1^, and each substrate exhibited a consistent spectral pattern (Figure 3a). Subsequently, the Raman intensity at 640 cm^−1^ was compared for the six substrates (Figure 3d), revealing excellent repeatability with an RSD value of 7.227%.

After reacting UA on a single AF-SERS substrate, we presented the measured Raman spectra for 50 random spots as a heat map image (Figure 3b). The bright line at 640 cm^−1^ in the image indicated the strong SERS intensity of UA on AF-SERS SERS substrates. Similarly, a comparison of Raman intensity at a specific UA peak of 640 cm^−1^ for each spot revealed excellent uniformity with an RSD value of 13.46% (Figure 3e).

Following the examination of various UA concentrations on the AF-SERS substrate, detection experiments were conducted for 10 concentrations, in the range from 1 mM to 1 nM, covering the normal physiological range of 200–500 μM (Figure 3c). As shown in Figure 3c, there is a noticeable tendency for the Raman intensity to increase with higher UA concentrations (10^0^–10^−5^ mM) at a specific Raman peak of 640 cm^−1^, aligning with the molecular structure of UA. The evaluation of the Raman intensity of UA at the specific Raman peak value of 640 cm^−1^ revealed a linear increase in the high concentration range, spanning from the normal physiological range (200–500 μM) to 1 mM (Figure 3f). The linear equation for UA concentration was calculated as I_HC_ = 211.9 X + 31,674 (R^2^ = 0.99) in the high concentration range. Additionally, a logarithmic linear increase was observed in the low concentration range (10 nM–100 μM), and the logarithmic linear equation for low concentration UA was calculated as I_LC_ = 9835 × log(X) − 22,636 (R^2^ = 0.987; X represents UA concentration (mM)). The LOD for UA was determined to be 1.16 nM using the following formula: LOD = 3.3 × standard deviation/slope of the regression curved line.

Based on these findings, we experimentally validated the ability of our AF-SERS substrate to detect UA concentrations within the normal range, as well as at deficient and excess concentrations. As indicated in Table S3, our results not only exhibit the highest sensitivity of the substrate in terms of LOD but also boast the broadest detection range compared to various UA detection methods, including electrochemical, fluorescence, and spectroscopic methods.

### 3.4. Detection Selectivity of UA Sensor with AF-SERS

**Figure 4:**
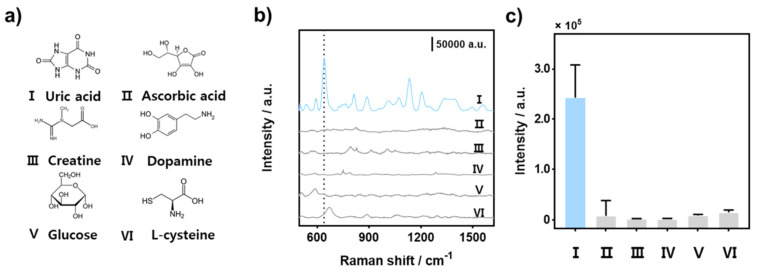
Selectivity analysis of the AF-SERS-based UA sensor. (**a**) Structural formula of selective groups: uric acid (I), ascorbic acid (II), creatine (III), dopamine (IV), glucose (V), and L-cysteine (VI). (**b**) SERS spectra and (**c**) SERS peak intensities at 640 cm^−1^ of UA and the selective groups.

To evaluate the selective UA detection capability of the fabricated AF-SERS substrate, signals from various metabolites were analyzed (Figure 4). Figure 4a presents the chemical formulations of target molecules used in the selectivity experiments, including UA (I), ascorbic acid (II), creatine (III), dopamine (IV), glucose (V), and L-cysteine (VI), which are representative metabolites [43,44,45,46]. UA was tested at a concentration of 800 μM, whereas the remaining substances were experimented with at a high concentration of 1 mM. The Raman spectrum results revealed a distinct and robust intensity at 640 cm^−1^, the UA-specific Raman peak, exclusively for UA. Other target molecules, even at high concentrations, did not exhibit peaks, which overlapped with UA (Figure 4b). Further analysis focused solely on the Raman intensity at 640 cm^−1^ demonstrated a value of 196,617 ± 18,102.54 for UA, whereas the remaining molecules displayed significantly lower values, consistently below 15,000 (ascorbic acid: 7559.80 ± 30,472.24, creatine: 1307.59 ± 917.42, dopamine: 917.57 ± 1525.35, glucose: 8298.72 ± 2247.93, and L-cysteine: 14,426.45 ± 4684.40) (Figure 4c). This rigorous comparison highlights the specificity of the proposed AF-SERS-based UA sensor, demonstrating its ability to selectively detect UA among other metabolites in the body, despite the relatively high concentrations of other target substances.

### 3.5. UA Detection in Real Sample: Human Serum and Urine

**Figure 5:**
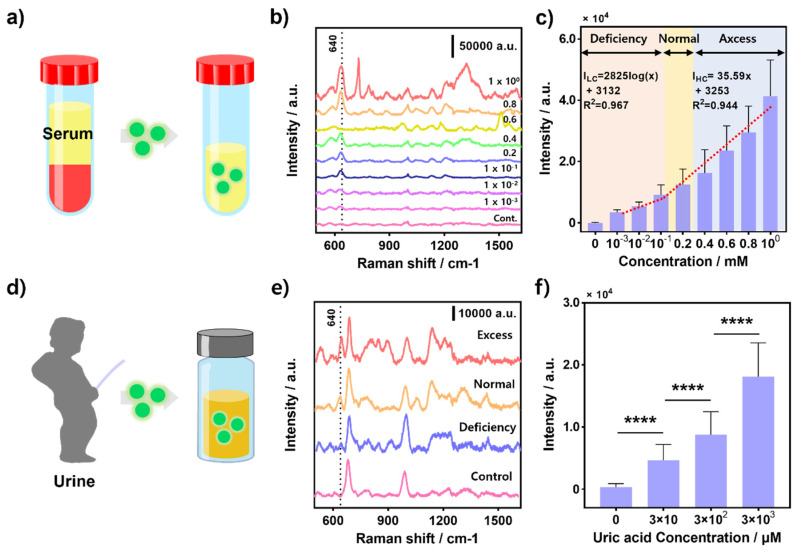
UA detection using the AF-SERS substrate in human serum and artificial urine. (**a**) Schematic of UA sampling in 10% diluted human serum. (**b**) SERS spectra and (**c**) SERS peak intensities at 640 cm^−1^ of 10% diluted human serum in UA in the concentration range of 1 μM–1 mM. (**d**) Schematic of UA sampling in urine. (**e**) SERS spectra and (**f**) SERS peak intensities at 640 cm^−1^ of various UA concentration ranges (deficiency, normal, excess) in urine. Each detection stage can be distinctly differentiated statistically (**** *p*-value < 0.0001).

Actual samples for detecting UA include human blood and urine. Therefore, to validate the detection capability of UA within the body using the AF-SERS-substrate-based UA sensor, we designed experiments for UA detection in human serum and real urine samples (Figure 5). First, for UA detection in serum, human serum was diluted at a 1:10 ratio (Figure 5a). Subsequently, UA was mixed into the diluted serum at various concentrations to prepare the samples. Samples were prepared from 1 mM to 1 μM, including a concentration of 200–500 µM, which corresponds to the normal range of UA in human serum. As shown in Figure 5b, the Raman signal at 640 cm^−1^ changes significantly as the concentration of UA changes. Specifically, the Raman intensity results by concentration at 640 cm^−1^ and specific Raman peak of UA are shown in Figure 5c. A log-linear increase is observed at low concentrations (1–100 μM), corresponding to conditions in which UA deficiency is anticipated. In contrast, a linear increase is observed at high concentrations (200 μM–1 mM), which are high-risk UA concentrations. The log-linear equation for low concentrations was calculated as I_LC_ = 2825 × log(X) + 3132 (R^2^ = 0.967), and the linear equation for high concentrations was calculated as I_HC_ = 35.59X + 3253 (R^2^ = 0.9644). Specifically, X denotes the concentration of UA (mM) in serum. The LOD of UA in serum was determined to be 1.05 μM using the LOD formula. The results indicate a decrease in sensitivity compared to the laboratory environment because various proteins in the serum act as interfering factors. However, despite this, the AF-SERS substrate exhibited detection performance, which far exceeded the normal range.

Following this, urine samples were prepared using synthetic human urine without UA (Figure 5d). Uric acid concentrations in the urine of healthy individuals typically range from 160 to 320 μM [47]. Therefore, we prepared a sample reflecting normal conditions by dissolving 300 μM of uric acid, within the normal range, in artificial urine. Additionally, urine under UA deficiency conditions was diluted to a concentration of 30 μM (1/10 of the normal range), and UA excess conditions were produced by adding UA to 3 mM (10 times beyond the normal range). Figure 5e presents the Raman spectra results for each urine sample. Artificial urine contains not only UA but also various proteins, especially urea. Furthermore, the presence of a Raman-specific peak (1000 cm^−1^) of urea was confirmed [48]. Among these, a specific peak related to UA appears at 640 cm^−1^, signifying that it is possible to detect normal UA levels, as well as deficiency and excess states, as shown in Figure 5f. Our UA detection sensor encompasses a broad spectrum within the normal range of uric acid, as illustrated in Figure S9, and in fact boasts a wider coverage compared to conventional detection range [49]. This level of detection capability, which identifies deficiencies and excesses based on UA concentration in urine samples, is also applicable to real urine samples in the future and is deemed suitable for potential application in future real-world field scenarios.

## 4. Conclusions

We developed an AF-SERS substrate for detecting UA by mimicking the ecological structure of a type of starfish. The leg and protrusion morphology of *Asterias forbesi* exhibited a highly enhanced Raman signal, and simulations confirmed that its structure possessed numerous hotspots. The AF-SERS substrate not only exhibited excellent SERS performance but also minimized oxidation-related damage, making it suitable for practical applications. Moreover, we proved the high repeatability and uniformity of the AF-SERS substrate for UA detection. Using the proposed AF-SERS substrate, we successfully detected UA in a laboratory environment down to a concentration of 1.16 nM and demonstrated selectivity against various metabolites. For real-world application of the sensor, we detected UA in human serum and actual urine samples. In serum, we could differentiate UA concentrations beyond the normal range, covering deficiencies and excesses, with a LOD of 1.05 μM. Similarly, we successfully detected UA in artificial urine samples corresponding to deficiency, normal, and excess states. In conclusion, our research confirms that the proposed AF-SERS substrate enables highly sensitive UA detection in real samples. Our findings suggest its potential as a practical detection platform for real-world UA applications.

## Data Availability

Data are contained within the article and Supplementary Materials.

## Supplementary Materials

The following supporting information can be downloaded at: https://www.mdpi.com/article/10.3390/bios14010008/s1. Figure S1: Optical images of a (a) SLNS-Ag and (b) SLNS-Au@Ag substrates; Figure S2: SEM-EDS data of AF-SESR substrate; Figure S3: (a) Raman spectrum of 100 μM R6G on only GNP and AF-SERS substrate respectively. (b) Raman intensity graph at 1508 cm^−1^, the specific Raman peak of R6G. (Scale bar: 500 nm); Figure S4: Raman spectral data of Au plate and SLNS-Ag substrate for R6G 100 μM. Upon reducing the y-axis scale in a specific region, the Raman signal of silver was identified at around 1000 cm^−1^ (indicated by the red square); Figure S5: (a) Raman spectrum of 100 μM R6G on only GNP and AF-SERS substrate respectively. (b) Raman intensity graph at 1508cm-1, the specific Raman peak of R6G; Figure S6: FEM-based electromagnetic simulation results for Au plate and RGB ratio spectrum of each area; Figure S7: Raman spectra of R6G for each day on AF-SERS substrate when exposed to harsh conditions (PBS buffer solution) for 7 days; Figure S8: Raman spectra of R6G for each day on GNP + SLNS-Ag substrate when exposed to harsh conditions (PBS buffer solution) for 7 days; Figure S9: A graph comparing the excretion concentration range of uric acid in the body using conventional technology, the normal range of uric acid, and the detection range of uric acid using AF-SERS substrate; Table S1: Comparison of enhancement factor performance of SERS substrates using various nanomaterial [37,44,50,51,52,53]; Table S2: Vibrational SERS band assignments for Uric acid [54,55]; Table S3: Comparison of the SERS sensing performances of our AF-SERS and other various sensing platform about UA [25,56,57,58,59,60,61,62,63].
