# Supplementary material for: Asterias forbesi-Inspired SERS Substrates for Wide-Range Detection of Uric Acid
Source: Biosensors (Basel). 2023 Dec 22;14(1):8. doi: 10.3390/bios14010008 (PMC10813034; doi:10.3390/bios14010008)
Supplement: Supplementary file 1 [file biosensors-14-00008-s001.zip › biosensors-2758697-supplementary.pdf]

biosensors

Supplementary Materials

Asterias forbesi-Inspired SERS Substrates for Wide-Range Detection of Uric Acid

Hyunjun Park [1,†], Kyunghwan Chai [1,†], Woochang Kim [1,†], Joohyung Park [1], Wonseok Lee [2,*] and Jinsung Park [1,*]

[1] Department of Biomechatronic Engineering, College of Biotechnology and Bioengineering, Sungkyunkwan University, Suwon 16419, Republic of Korea
[2] Department of Electrical Engineering, Korea National University of Transportation, Chungju 27469, Republic of Korea
[*] Correspondence: wslee@ut.ac.kr (W.L.); nanojspark@skku.edu (J.P.)
[†] These authors contributed equally to this study.

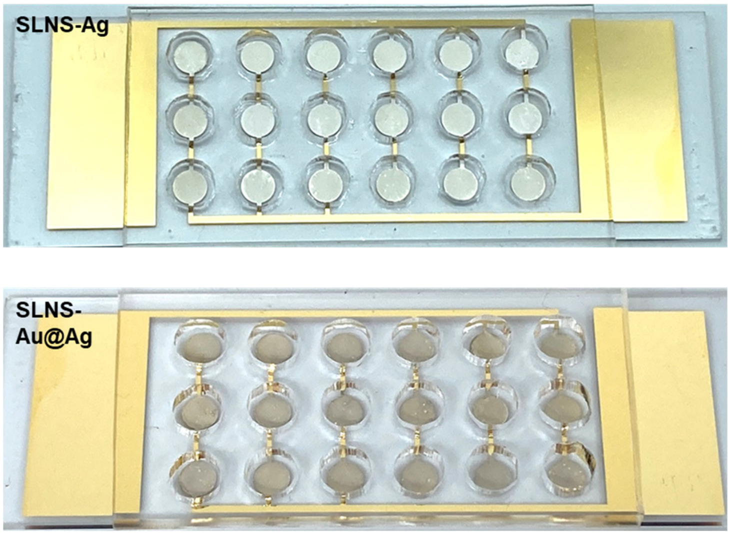

Figure S1. Optical images of a (a) SLNS-Ag and (b) SLNS-Au@Ag substrates.

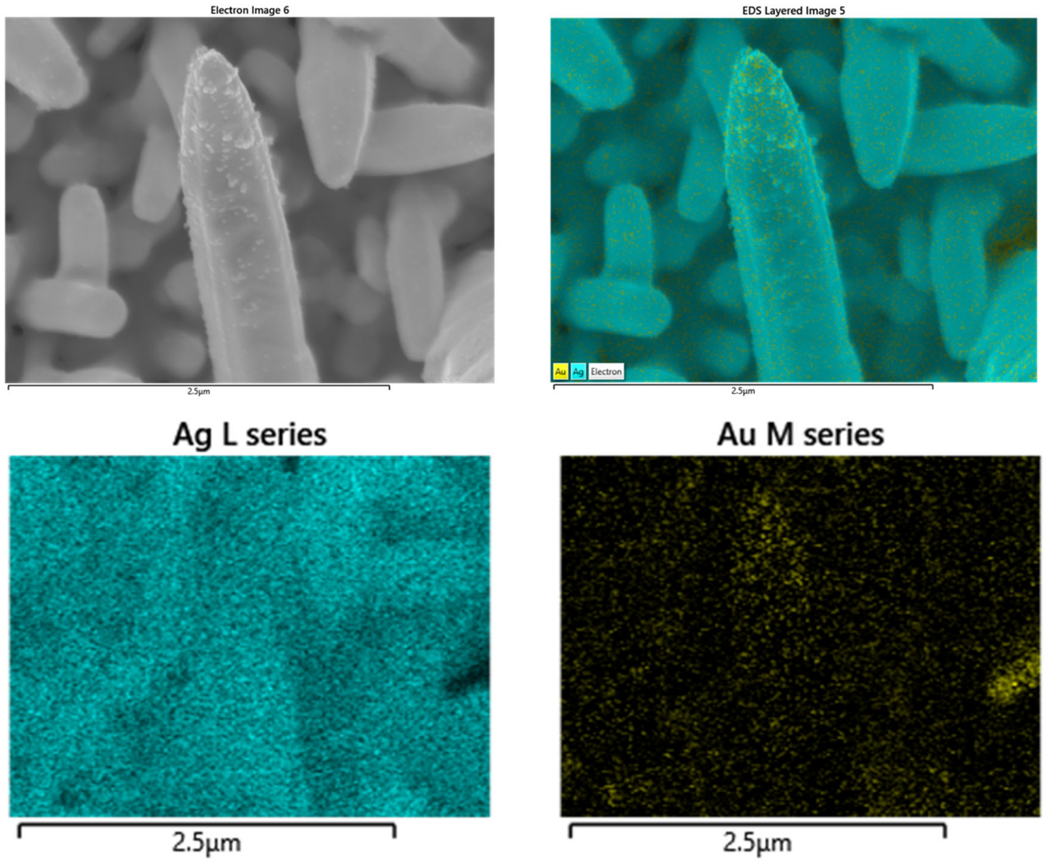

Figure S2. SEM-EDS data for AF-SESR substrate.

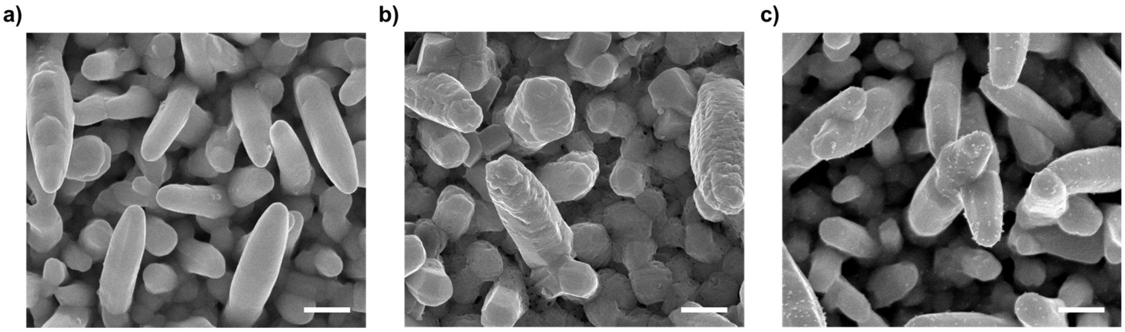

Figure S3. a) Raman spectrum of 100 μM R6G on GNP-only and AF-SERS substrate, respectively. b) Raman intensity graph at 1508 cm-1, the specific Raman peak of R6G (Scale bar: 500 nm).

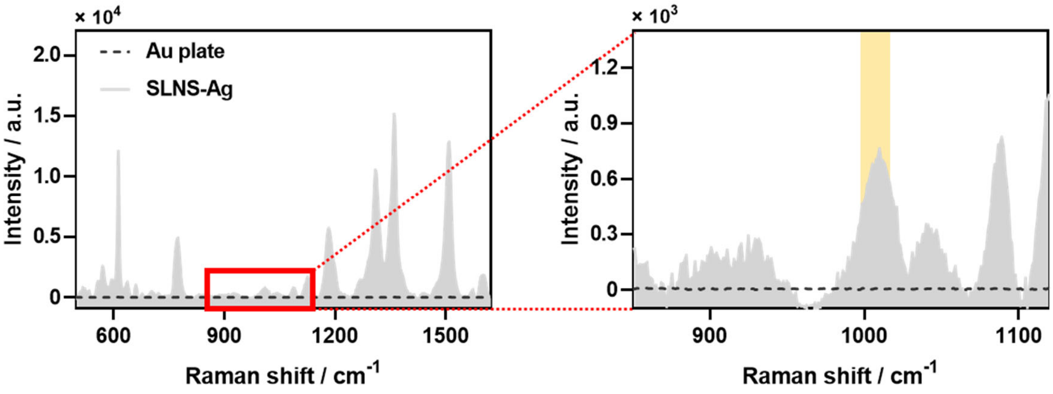

Figure S4. Raman spectral data of Au plate and SLNS-Ag substrate for R6G 100 μM. Upon reducing the Y-axis scale in a specific region, the Raman signal of silver was identified at around 1000 cm^{-1} (indicated by the red square).

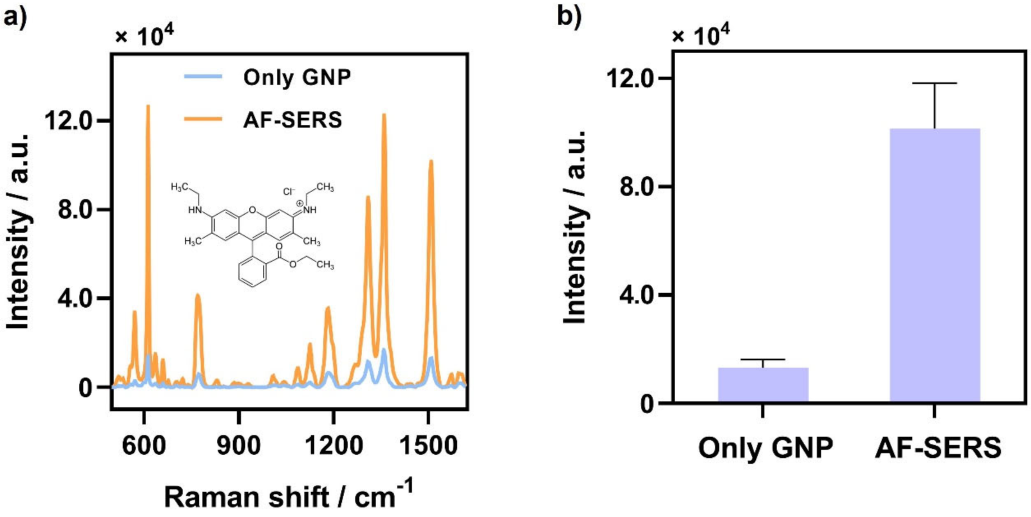

Figure S5. a) Raman spectrum of 100 μM R6G on GNP-only and AF-SERS substrate, respectively. b) Raman intensity graph at 1508 cm^{-1}, the specific Raman peak of R6G.

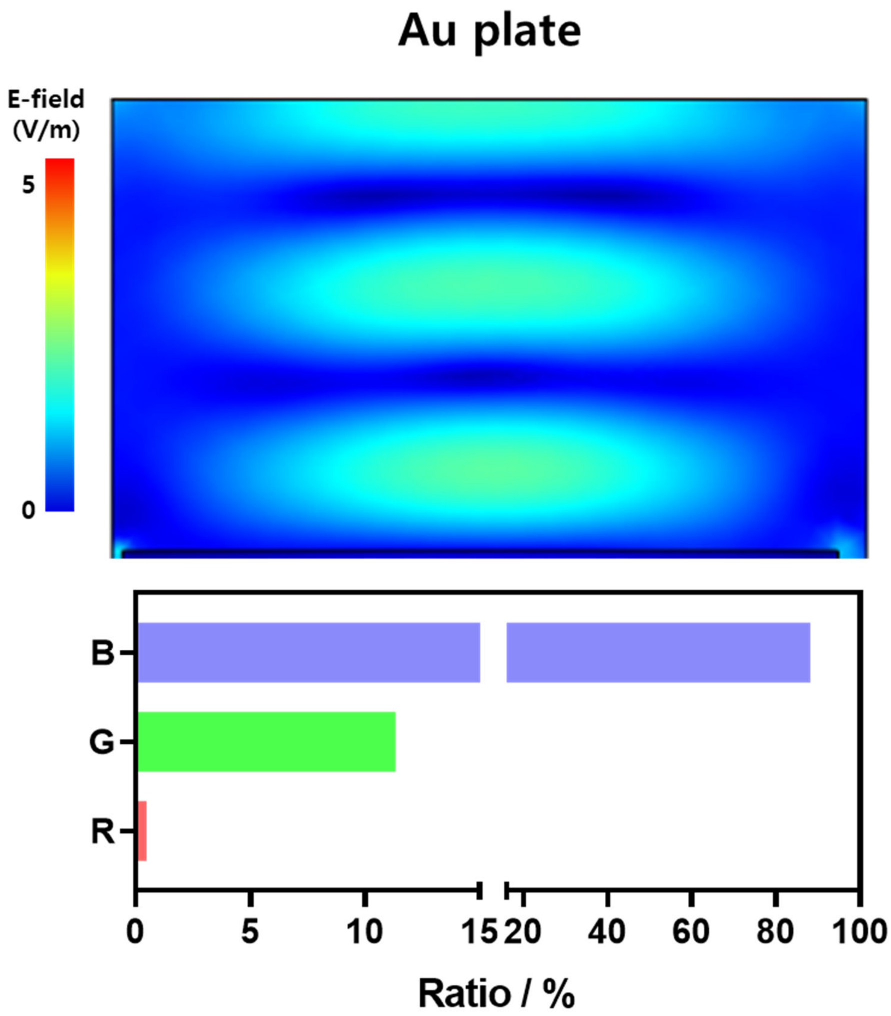

Figure S6. FEM-based electromagnetic simulation results for Au plate and RGB ratio spectrum of each area.

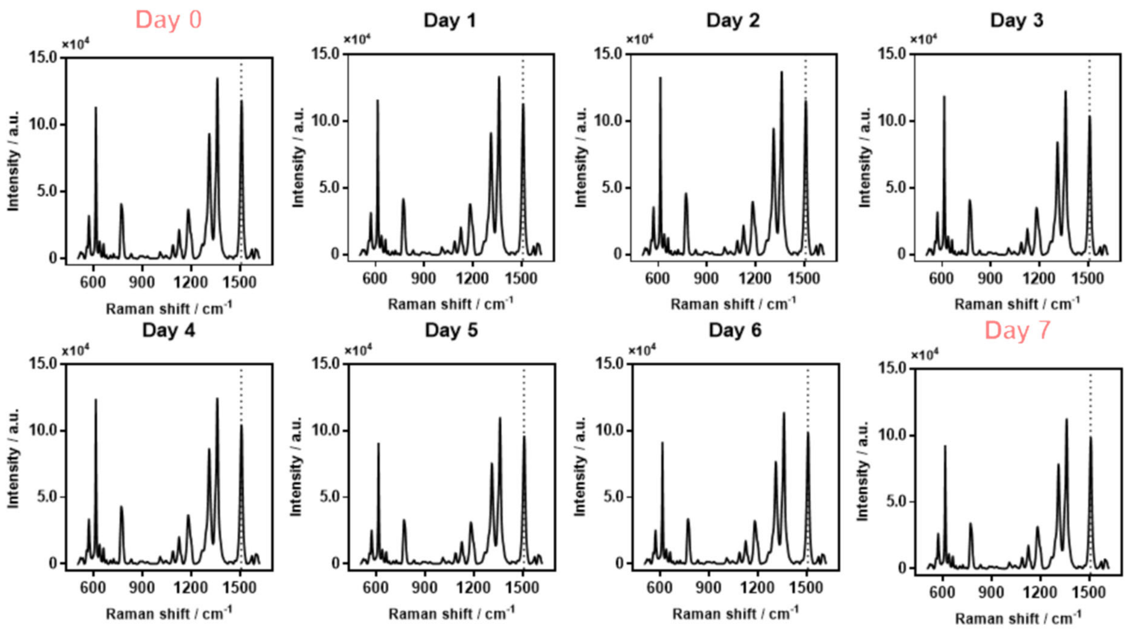

Figure S7. Raman spectra of R6G for each day on AF-SERS substrate when exposed to harsh conditions (PBS buffer solution) for 7 days.

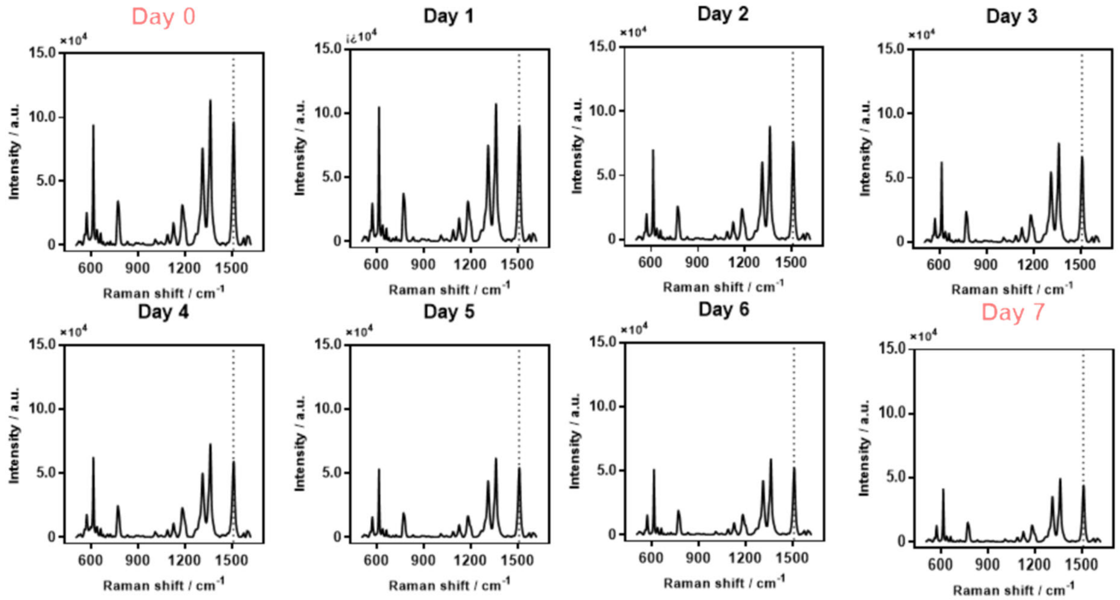

Figure S8. Raman spectra of R6G for each day on GNP + SLNS-Ag substrate when exposed to harsh conditions (PBS buffer solution) for 7 days.

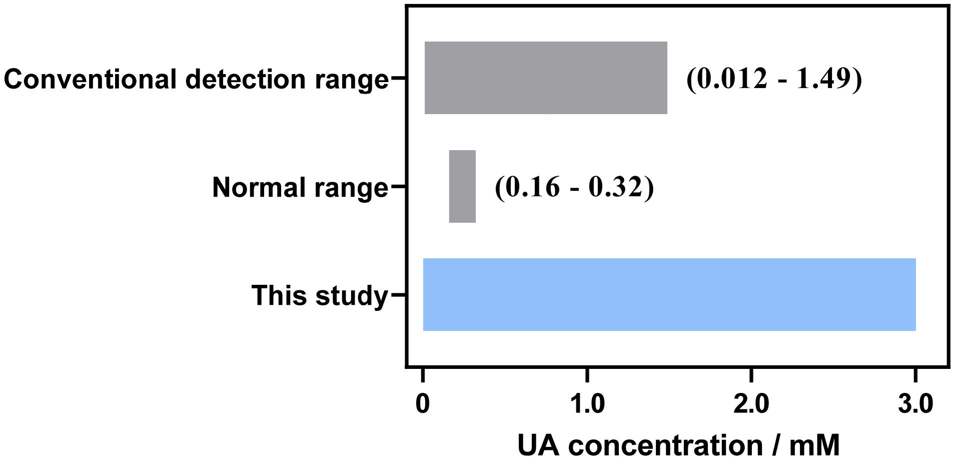

Figure S9. A graph comparing the excretion concentration range of uric acid in the body using conventional technology, the normal range of uric acid, and the detection range of uric acid using AF-SERS substrate.

Table S1. Comparison of enhancement factor performance of SERS substrates using various nanomaterials.

Year	Substrate	Enhancement Factor	Reference
2020	Au nanocone	8.5×10^6	[1]
2021	Au@Ag hollow nanocubes	7.52×10^9	[2]
2022	Opal structure	4.22×10^4	[3]
2023	Octahedral oxygen vacancy $MnCo_2O_4/Ag$ ($VO\text{-}MnCo_2O_4/Ag$)	2.7×10^6	[4]
2023	CdSe quantum dot	5.05×10^6	[5]
2024	3D nanosilver trees/sea-urchin-like gold	1.9×10^7	[6]
**2023**	**AF-SERS**	$\mathbf{3.658 \times 10^{11}}$	**This work**

Table S2. Vibrational SERS band assignments for uric acid.

Raman Shift / cm^{-1}	Vibrational Band Assignment[7,8]
508 (vwsh)	C-N-C ring vibrations
534 (vw)	
592 (vw)	Ring breathing mode
640 (s)	Skeletal ring deformation
730 (w)	N-H bending
812 (m)	Ring vibration
889 (m)	N-H bending
1017 (w)	Ring vibration
1134 (vs)	C-N
1206 (m)	N-C-C stretching and bending
1554 (m)	C-N stretching

Table S3. Comparison of SERS sensing performances of our AF-SERS and various other sensing platforms for UA.

Detection Method	Detection Range	Detection Limit	Reference
Electrochemical	800 pM–241 µM	300 pM	[9]
Electrochemical	100 nM–30 µM	23 nM	[10]
Electrochemical	4–200 µM	1.36 µM	[11]
Fluorescence	10–100 µM	1.2 µM	[12]
Fluorescence	0.2–150 µM	0.05 µM	[13]

Fluorescence	10–100 µM	1.214 µM	[14]
SERS	5 µM–1 mM	1.7 µM	[15]
SERS	0.2–1000 µM	0.1 µM	[16]
SERS	20–100 µM	20 µM	[17]
**SERS**	**10 nM–1 mM**	**1.18 nM**	**This work**

References

1. Huang, C.Y.; Hsiao, H.C. Integrated EC-SERS Chip with Uniform Nanostructured EC-SERS Active Working Electrode for Rapid Detection of Uric Acid. *Sensors 2020, Vol. 20, Page 7066* **2020**, *20*, 7066, doi:10.3390/S20247066.
2. Bhattacharjee, G.; Majumder, S.; Senapati, D.; Banerjee, S.; Satpati, B. Core-Shell Gold @silver Hollow Nanocubes for Higher SERS Enhancement and Non-Enzymatic Biosensor. *Mater Chem Phys* **2020**, *239*, 122113, doi:10.1016/J.MATCHEMPHYS.2019.122113.
3. Li, J.; Cui, X.; Yang, X.; Qiu, Y.; Li, Y.; Cao, H.; Wang, D.; He, W.; Feng, Y.; Yang, Z. Quantification of Uric Acid Concentration in Tears by Using PDMS Inverse Opal Structure Surface-Enhanced Raman Scattering Substrates: Application in Hyperuricemia. *Spectrochim Acta A Mol Biomol Spectrosc* **2022**, *278*, 121326, doi:10.1016/J.SAA.2022.121326.
4. Tan, Y.; Qi, M.; Jiang, H.; Wang, B.; Zhang, X. Determination of Uric Acid in Serum by SERS System Based on VO-MnCo2O4/Ag Nanozyme. *Anal Chim Acta* **2023**, *1274*, 341584, doi:10.1016/J.ACA.2023.341584.
5. Wang, K.; Chen, Z.; Li, Y.; Zhang, Y. Top-down Produced CdSe Quantum Dots as an Ultrasensitive SERS Platform for the Detection of Uric Acid. *Mater Chem Front* **2023**, *7*, 1624–1632, doi:10.1039/D2QM01275H.
6. Lin, X.; Li, L.J.; Guo, H.Y.X.; Li, R.; Feng, J. Preparation of 3D Nano Silver Trees/Sea Urchin-like Gold and SERS Detection of Uric Acid. *Spectrochim Acta A Mol Biomol Spectrosc* **2024**, *305*, 123464, doi:10.1016/J.SAA.2023.123464.
7. Westley, C.; Xu, Y.; Carnell, A.J.; Turner, N.J.; Goodacre, R. Label-Free Surface Enhanced Raman Scattering Approach for High-Throughput Screening of Biocatalysts. **2016**, doi:10.1021/acs.analchem.6b00813.
8. Goodall, B.L.; Robinson, A.M.; Brosseau, C.L. Electrochemical- Surface Enhanced Raman Spectroscopy (E-SERS) of Uric Acid : A Potential Rapid Diagnostic Method for Early Preeclampsia Detection. *Physical Chemistry Chemical Physics* **2013**, *15*, 1382–1388, doi:10.1039/C2CP42596C.
9. Kokulnathan, T.; Wang, T.J.; Kumar, E.A.; Duraisamy, N.; An-Ting Lee An Electrochemical Platform Based on Yttrium Oxide/Boron Nitride Nanocomposite for the Detection of Dopamine. *Sens Actuators B Chem* **2021**, *349*, 130787, doi:10.1016/J.SNB.2021.130787.
10. Zhu, X.; Xuan, L.; Gong, J.; Liu, J.; Wang, X.; Xi, F.; Chen, J. Three-Dimensional Macroscopic Graphene Supported Vertically-Ordered Mesoporous Silica-Nanochannel Film for Direct and Ultrasensitive Detection of Uric Acid in Serum. *Talanta* **2022**, *238*, 123027, doi:10.1016/J.TALANTA.2021.123027.
11. Wang, H.; Xie, A.; Li, S.; Wang, J.; Chen, K.; Su, Z.; Song, N.; Luo, S. Three-Dimensional g-C3N4/MWNTs/GO Hybrid Electrode as Electrochemical Sensor for Simultaneous Determination of Ascorbic Acid, Dopamine and Uric Acid. *Anal Chim Acta* **2022**, *1211*, 339907, doi:10.1016/J.ACA.2022.339907.
12. Zhao, X.Y.; Yang, Q.S.; Wang, J.; Fu, D.L.; Jiang, D.K. A Novel 3D Coordination Polymer Constructed by Dual-Ligand for Highly Sensitive Detection of Purine Metabolite Uric Acid. *Spectrochim Acta A Mol Biomol Spectrosc* **2021**, *262*, 120065, doi:10.1016/J.SAA.2021.120065.
13. Li, F.; Chen, J.; Wen, J.; Peng, Y.; Tang, X.; Qiu, P. Ratiometric Fluorescence and Colorimetric Detection for Uric Acid Using Bifunctional Carbon Dots. *Sens Actuators B Chem* **2022**, *369*, 132381, doi:10.1016/J.SNB.2022.132381.
14. Sumalatha, V.; Anujya, C.; Balchander, V.; Dhanalaxmi, B.; Pradeep Kumar, M.; Ayodhya, D. Hydrothermal Fabrication of N-CeO2/p-CuS Heterojunction Nanocomposite for Enhanced Photodegradation of Pharmaceutical Drugs in Wastewater under Visible-Light and Fluorometric Sensor for Detection of Uric Acid. *Inorg Chem Commun* **2023**, *155*, 110962, doi:10.1016/J.INOCHE.2023.110962.
15. Lu, D.; Cai, R.; Liao, Y.; You, R.; Lu, Y. Two-Dimensional Glass/p-ATP/Ag NPs as Multifunctional SERS Substrates for Label-Free Quantification of Uric Acid in Sweat. *Spectrochim Acta A Mol Biomol Spectrosc* **2023**, *296*, 122631, doi:10.1016/J.SAA.2023.122631.
16. Jiang, L.; Wang, L.; Zhan, D.S.; Jiang, W.R.; Fodjo, E.K.; Hafez, M.E.; Zhang, Y.M.; Zhao, H.; Qian, R.C.; Li, D.W. Electrochemically Renewable SERS Sensor: A New Platform for the Detection of Metabolites Involved in Peroxide Production. *Biosens Bioelectron* **2021**, *175*, 112918, doi:10.1016/J.BIOS.2020.112918.
17. Durai, L.; Badhulika, S. A Wearable PVA Film Supported TiO2 Nanoparticles Decorated NaNbO3 Nanoflakes-Based SERS Sensor for Simultaneous Detection of Metabolites and Biomolecules in Human Sweat Samples. *Adv Mater Interfaces* **2022**, *9*, 2200146, doi:10.1002/ADMI.202200146.
